# Comparing net returns in the feedlot: *Bos Taurus* vs. *Bos Indicus* influenced steers with varying anabolic implant intensity

**DOI:** 10.1093/tas/txac111

**Published:** 2022-08-21

**Authors:** Ryan Feuz, Caleb C Reichhardt, Ryan Larsen, Kara J Thornton, Mathew D Garcia

**Affiliations:** Applied Economics Department, Utah State University, Logan, UT 84322, USA; Department of Human Nutrition, Food and Animal Sciences, University of Hawaiʻi at Mānoa, Honolulu, HI 96822, USA; Applied Economics Department, Utah State University, Logan, UT 84322, USA; Animal, Dairy and Veterinary Science Department, Utah State University, Logan, UT 84322, USA; Animal, Dairy and Veterinary Science Department, Utah State University, Logan, UT 84322, USA

**Keywords:** anabolic implants, santa gertrudis, SERF analysis

## Abstract

There are two main beef cattle breed types: *Bos Taurus* (BT) and *Bos Indicus* (BI). Past research has demonstrated various expected differences in growth, temperament, feeding behavior, and carcass characteristics between these breed types when administered varying levels of anabolic implant. However, little is known about the differences in expected economic returns between these cattle types. The objective of this research is to simulate and compare the expected net returns of BT, Angus (AN) steers and BI influenced, Santa Gertrudis (SG) steers, with moderate or high intensity levels of implants relative to a control with no implant. The animal performance and carcass data for this economic analysis was provided from a recent feeding experiment of AN and SG influenced steers. In the experiment, sixty steers were stratified by weight and breed in a 2 × 3 factorial design examining the two different breeds: AN (*N* = 38) or SG influenced (*N* = 22), and three implant strategies: no implant (*N* = 20), a moderate intensity implant protocol (d0 implant: Revalor-G, d56 implant: Revalor-IS, d112 implant: Revalor-S; n=20), or a high intensity implant protocol (d0 implant: Revalor-IS, d56 implant: Revalor-S, d112 implant: Revalor-200; *N* = 20). The steers performance and carcass data were used together with publicly available price and input costs data in the simulation of net returns per animal for each of the treatment groups. Results demonstrated that both moderate and high intensity implanted BT steers have higher expected net return (US$78.70/hd. and US$75.84/hd., respectively) compared to BI moderate and high intensity implanted steers (US$47.03/hd. and $6.98/hd., respectively). Stochastic efficiency analysis with respect to a function demonstrated when certainty equivalent values are constrained to those ≥US$0, only the moderate implanted BT steers would be included in the efficient set.

## INTRODUCTION

There are two main beef cattle breed types: *Bos Taurus* (BT) and *Bos Indicus* (BI) (Coles et al., 2014). The BT breed type currently dominates the U.S. cattle industry with BI-influenced cattle representing only 8% of the total U.S. cowherd (Cundiff et al., 2012). BT genetics have gained favor for their recognized superior carcass traits, early sexual maturation, and docile temperament. Comparatively, BI cattle have been shown to have a more excitable temperament—increasing management difficulty, and leading to decreased meat tenderness, lower marbling percentages, and less favorable palatability characteristics (Crouse et al., 1989; Voisinet et al., 1997; Wright et al., 2018). However, BI influenced cattle tend to have a shorter, smooth coat of hair aiding in their ability to better withstand higher temperatures (Forbes et al., 1998). Additionally, BI cattle have been shown to consume less water, adapt better to nutritional stress, and resist parasites (Winchester and Morris, 1956; Forbes et al., 1998). These positive traits have long been exploited while simultaneously mitigating concerns of the less favorable traits through cross breeding with BT cattle. Aside from improving genetics through cross breeding, cattle producers often also use growth-promoting technologies, such as anabolic implants, to increase production (Capper and Hayes, 2012). Anabolic implants are routinely used to improve efficiency and growth of cattle by 15% to 20%, leading to increased economic returns to producers (Duckett and Pratt, 2014).

Reichhardt et al. (In Review) conducted a feeding trial, wherein they sought to determine the optimal anabolic implant protocols of BI influenced cattle in temperate climates compared to BT cattle raised in temperate climates. Santa Gertrudis (SG) influenced steers were used as the BI influenced cattle and were compared to Angus (AN). SG cattle are a cross between BI and BT cattle genetics and are a result of crossing Brahman (BI) and Shorthorn (BT) breeds (3/8 Brahman and 5/8 Shorthorn). Within the feeding trial of Reichhardt et al. (In Review), the BI influenced steers were 19% Brahman, 31% Shorthorn, and 50% Angus. The steers were placed randomly into pens equipped with GrowSafe bunks, fed the same ration, and weighed and ultrasounded at consistent intervals. Dry matter intake, feeding behavior, and carcass data were collected and backfat measurements and weights were recorded. The authors found that total average daily gain was increased (*P* < 0.0001) by 29.4% in ‘high intensity’ (HI) implanted steers compared to control steers with no implant (CON), while it was increased by 26% in the ‘moderate’ implant (MI) steers compared to CON steers. Hot carcass weight (HCW) was altered by treatment with MI and HI steers having larger (*P* < 0.0002) carcasses than CON steers, with the carcasses being 12.8% and 14.8% heavier, respectively. Marbling score was influenced by breed (*P* = 0.001), with AN steers having improved (*P* = 0.001) marbling compared to SG-sired steers. These differences resulted in an estimated US$51 decrease in net return per head, on average, for the SG steers as compared to the AN steers in their study. Additionally, the moderate intensity implant protocol was estimated to increase net return per head, on average, by US$97.28, regardless of breed, while the high intensity implant protocol increased net return by only US$80.84. The researchers concluded that regardless of breed type, a moderate intensity anabolic implant protocol is optimal for steers raised in a temperate climate.

While the average changes to expected economic returns per head estimated by Reichhardt et al. (In Review) were informative, they did not provide a complete analysis focused on the expected economic returns of the BT and BI influenced steers within their study. Therefore, the objective of this study is to provide an evaluation of key economic drivers in the comparison of BI-influenced and BT cattle with varying levels of anabolic implant intensity. Specifically, this study will: (1) take a simulation approach using the data from the Reichhardt et al. (In Review) study to provide a more complete risk profile in the discussion of differences in expected net returns per head; (2) determine break-even values in specific grid pricing premiums or discounts needed for the expected net returns for the BI-influenced and BT cattle within the study to have equivalent expected net returns; (3) provide a qualitative discussion as to when introduction of BI genetics may be the profitable decision for feedlots under certain circumstances.

## MATERIALS AND METHODS

### Animals, Experimental Design, and Treatments

The experiment was conducted at the Utah State University feedlot and used a 3 × 2 factorial design. The steers were initially stratified by weight at the start of the experiment. There were a total of 60 steers, 38 AN sired (590.0 ± 12.13 lb.) and 22 SG sired (621.9 ± 15.0 lb.). None of the steers had previously received any growth promotants. Both the AN and SG steers were out of commercial Angus dams. At the onset of the trial, steers were given electronic and visual ear tags and then assigned to one of three anabolic implant treatments: 1) no implant (**CON**; *N* = 20), 2) a moderate intensity implant protocol (d0 implant: Revalor-G, d56 implant: Revalor-IS, d112 implant: Revalor-S; **MI**; *N* = 20), or 3) a high intensity implant protocol (d0 implant: Revalor-IS, d56 implant: Revalor-S, d112 implant: Revalor-200; **HI**; *N* = 20). The steers were then placed randomly into one of four covered pens each equipped with two GrowSafe feed-bunks with free choice access to water. Each of the pens housed 15 steers. The steers were all fed the same diet. The initial background diet consisted of 44.5% (DM basis) concentrate. Following the background diet, subsequent diets were stepped up in DM basis concentrate over a 35-d period between 10% and 12% (DM basis) concentrate every 10 d culminating with the finishing diet consisting of 81% (DM basis). A summary of key results from Reichhardt et al. (In Review) is contained within Table 1. For additional information regarding the rations fed during the trial and for a complete discussion of results contained within Table 1, reference Reichhardt et al. (In Review).

**Table 1.** Summary of comparative results between AN and SG steers in Reichhardt et al. (In Review) study

	Treatment groups^1^						SEM	*P*-values^2^		
	AN-CON	AN-MI	AN-HI	SG-CON	SG-MI	SG-HI		Breed	Trt	B X T
Steers (n)	13	12	13	7	8	7				
Total average DMI (lbs./day)	18.67	22.49	23.17	22.20	23.99	24.49		0.007	<0.0001	0.093
Total G:F	0.13	0.19	0.15	0.16	0.28	0.14		0.57	0.007	0.43
Total ADG (lbs./day)	2.73	3.38	3.37	2.84	3.64	3.53	0.20	0.39	<0.0001	0.35
Total gain (lbs.)	529.11	644.19	699.09	544.10	698.42	659.18	45.86	0.68	<0.0001	0.26
Dressing percentage	59.6	61.2	61.1	60.11	60.0	60.6	10	0.56	0.43	0.61
Hot carcass weight (lbs.)	296.04	342.52	357.86	321.87	354.26	351.47	12.7	0.26	0.0002	0.35
Marbling score	398.96	374.78	372.31	343.54	328.81	323.71	19.9	0.001	0.37	0.96
Cold camera yield grade	2.75	2.95	2.98	3.14	3.04	2.99	0.26	0.39	0.97	0.69

Steers were assigned to one of three implant treatments: (1) no implant (CON; *N* = 20), (2) a moderate intensity implant protocol (d0 implant: Revalor-G, d56 implant: Revalor-IS, d112 implant: Revalor-S; MI; *N* = 20), or (3) a high intensity implant protocol (d0 implant: Revalor-IS, d56 implant: Revalor-S, d112 implant: Revalor-200; HI; *N* = 20) and of two different breed types Angus (AN) or Santa Gertrudis influenced (SG). ^2^*P*-values indicate the effect of Breed, Treatment (TRT), or B × T (Breed × Treatment).

### Economic Methods

Feedlots typically operate on thin profit margins per head (Henderson, 2019). If feedlot managers are to be enticed into feeding BI influenced cattle, certainly expected profit margins would weigh heavily in the decision process. The results contained within Table 1 demonstrate that there are key differences within the treatment groups that would be expected to affect the net returns which motivates an economic analysis of the treatment groups. The economic analysis methods for livestock research trials and the reporting of the methodology have been shown to vary greatly and be inconsistent (Dixon, 2022). Thus, we aim to clearly define the economic analysis methodology used to compare the treatments within this trial. To compare the economic performance of the six treatment groups (AN-CON, AN-MI, AN-HI, SG-CON, SG-MI, and SG-HI) within this study, separate budgets (enterprise analysis) were created for each group from which the expected net return above feed and implant costs per head was calculated and compared across groups. The budgets for each treatment calculated the expected net revenue as the sales revenue of fed animals less the purchase price of feeder animals. Each budget then considered feed and treatment variable costs that consisted of the individual feed components of the various rations as well as the cost of the implant if applicable. These variable costs were then subtracted from the net revenue to arrive at an expected net return above feed and treatment cost per treatment. The expected net return per head (revenue – feed and implant costs) was calculated as


$$\begin{array}{l} NR_{i}=\frac{DP_{i}(DW_{i})}{100}-\frac{FP_{i}(IW)}{100}-FC_{i}-IC_{i} \end{array}$$ (1)

where *NR*_*i*_ is the net return ($/head) of treatment group *i*, *DP*_*i*_ is the dressed price ($/cwt) of treatment group *i*, *DW*_*i*_, is the dressed weight (lbs.) of treatment group *i*, *FP*_*i*_, is the feeder cattle price ($/cwt) of treatment group *i*, *IW* is the initial weight (lbs.), *FC*_*i*_ is the total feed cost for treatment group *i*, and *IC*_*i*_ is the total implant cost for treatment group *i*.

Within Reichhardt et al. (In Review), the average net return per head per treatment group was calculated and compared. Using treatment group average values in the calculation of NR was an adequate starting point in the economic comparison of the treatment groups. However, additional insight can be gained by taking a simulation approach to calculate *NR*_*i*_. For this study, equation 1 was updated to allow key variables to vary stochastically. *NR*_*i*_ was then simulated over 10,000 iterations to provide a more complete risk profile for expected net return per treatment group. All simulation was conducted using Palisades @Risk Decision Tools Suite 7.6 (2019).

The dressed price (*DP*_*i*_) was calculated using grid pricing. The base value varied stochastically by fitting a distribution to the past five years of historic dressed prices using the five-market average dressed price (LMIC, 2021a). Grid pricing premiums and discounts were then added to the base value according to stochastically determined USDA quality and yield grades for each treatment group (distributions fit to observed treatment group quality and yield grades). The grid premiums and discounts used in the simulation were taken from the “National weekly direct slaughter cattle - premiums and discounts” report dated August 9, 2021. Prime was valued at $19.54/cwt. above Choice, while Select and Standard were discounted –US$17.92 and –US$31.50/cwt. respectively. Yield grades 1 and 2 were valued at US$3.69 and US$1.58/cwt. above yield grade 3, while yield grades 4 and 5 were discounted – US$11.23 and – US$16.85/cwt, respectively (reference Table 2).

**Table 2.** Hypothetical break-even price grid for simulated difference of net return between AN-MI and SG-MI compared with original price grid

Quality	Original value^1^	Break-even value^2^
Prime	US$19.54	US$19.54
Choice	US$0.00	US$0.00
Select	–US$17.92	–US$17.31
Standard	–US$31.50	–US$22.65
Yield		
Grade 1	US$3.69	US$14.90
Grade 2	US$1.58	US$6.71
Grade 3	US$0.00	US$0.00
Grade 4	–US$11.23	–US$11.23
Grade 5	–US$16.85	–US$16.85

The original price grid is the price grid used in the simulation analysis with the results displayed in Table 2. The grid values were taken from the “National weekly direct slaughter cattle – premiums and discounts” report dated August 9, 2021.

The hypothetical break-even price grid was obtained through a dynamic optimization model (Palisades @Risk Decision Tools Suite, 2019) to set the simulated net return of AN-MI equal to net return or SG-MI.

The feeder cattle price for AN cattle was held constant at US$157.00/cwt. (past five years average of the Colorado combined auction prices for feeder steers 550 to 650 lbs, LMIC, 2021b). The stocker price for the SG cattle was discounted US$3.50/cwt. (US$153.50) to coincide with findings in the literature (Schroeder et al. 1988; Feuz et al., 2008; Hawkes et al., 2008; Troxel et al., 2011) that support discounts of this approximate size for BI influenced feeder cattle. Auction buyers often discount based on assumed BI influence identified through visual identification at the time of sale of BI traits such as droopy ears, humped back, or loose skin. If BI influenced cattle exhibit no distinguishable characteristics the stocker price would not be discounted relative to BT cattle.

Using Superior Livestock Auction data, Hawkes et al. (2008) developed hedonic pricing models to estimate the discounts associated with BI breeds. They estimated a discount of US$3.22/cwt. for lots comprised of BI influence cattle. Feuz et al. (2008) also used Superior Livestock Auction data and estimated a US$5.00/cwt. discount for English × Exotic × Ear feeder cattle relative to Angus, with “Ear” representing BI influence.

Troxel et al. (2011) used the Arkansas Livestock Market Survey to report discounts associated with different characteristics of feeder cattle. SG was not reported as a breed type, however discounts for Brahman (another common BI breed) influenced feeder cattle were reported. Discounts for 1/4 Brahman cross and Angus × ¼ Brahman were reported as US$3.34/cwt and US$1.39/cwt, respectively. Schroeder et al. (1988) used Kansas feeder cattle auction data and estimated discounts for Brahman <1/4 influenced and Brahman >1/4 influenced feeder cattle of US$1.75/cwt and US$7.05/cwt, respectively. Based on the range of estimates of discounts for the varying levels of BI influenced feeder cattle collected from these studies, we assumed the US$3.50 discount for the SG influenced steers in our study.

The initial weight for each treatment group was assumed constant at 600 lbs., such that the input cost to the feedlot for stockers would be consistent except for the US$3.50/cwt. discount in price for the SG steers. The dressed weight was calculated as the initial weight plus the product of days on feed (DOF), treatment group ADG, and the treatment group dressing percentage. Within the calculation of dressed weight both ADG and dressing percentage varied stochastically using triangle distributions with the parameters of the distribution informed by the actual feeding trial data for each treatment group, while DOF was assumed constant at 190.5 d (length of feeding trial). The costs for individual ration feed components (alfalfa, haylage, corn, etc.) for each treatment group were stochastically determined through fitted distributions of 5-yr feed prices ($/lb.) multiplied by the total pounds of intake as measured by the GrowSafe feed bunks (varying stochastically) and the corresponding percentage of the feed components within the feed ration. The distributions used within the simulation are described in Table A1 and the “*Distribution*” section of Appendix 1.

## RESULTS AND DISCUSSION

**Figure 1.**
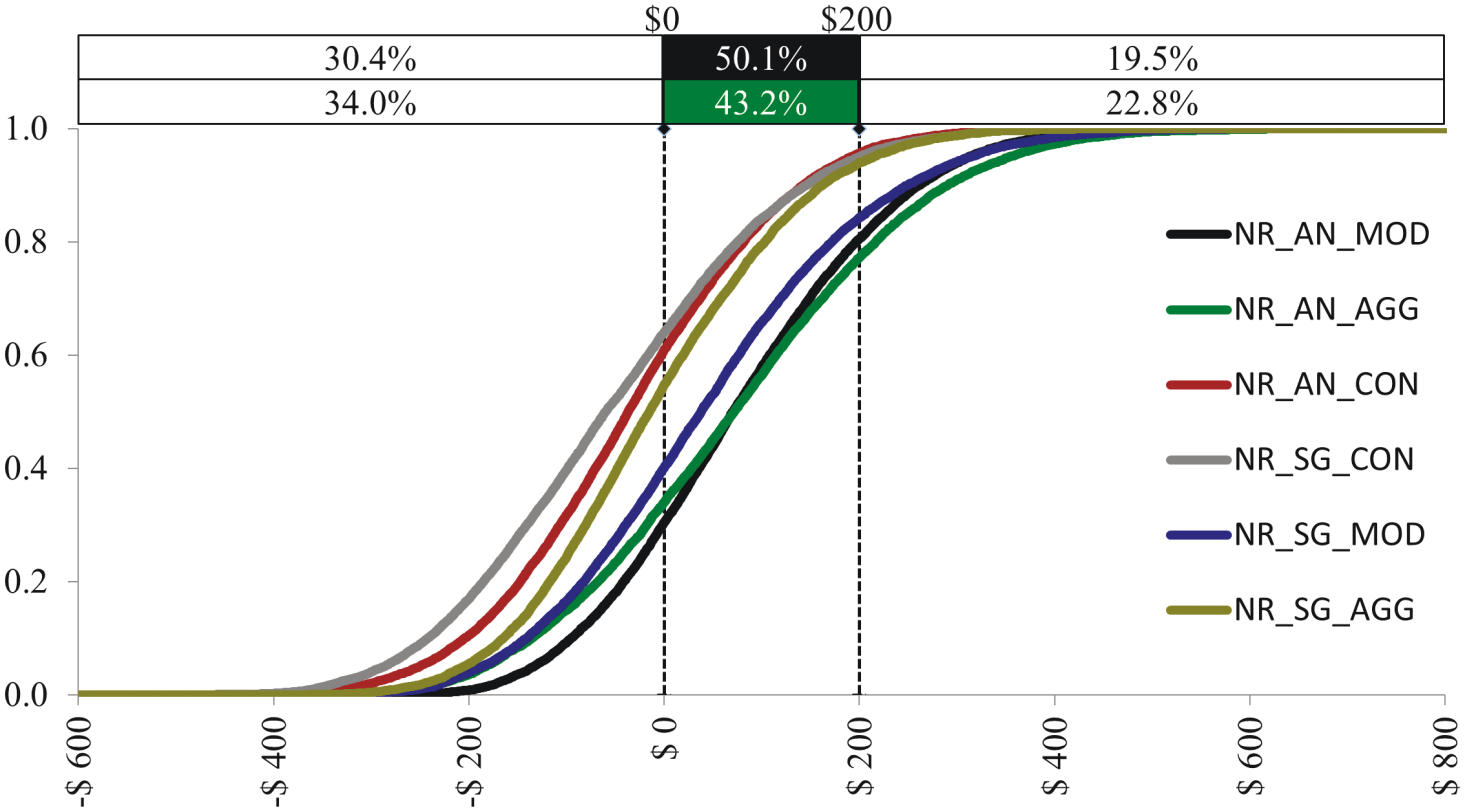
Simulated Cumulative Distribution Functions for each Treatment Group Net Return.

**Figure 2.**
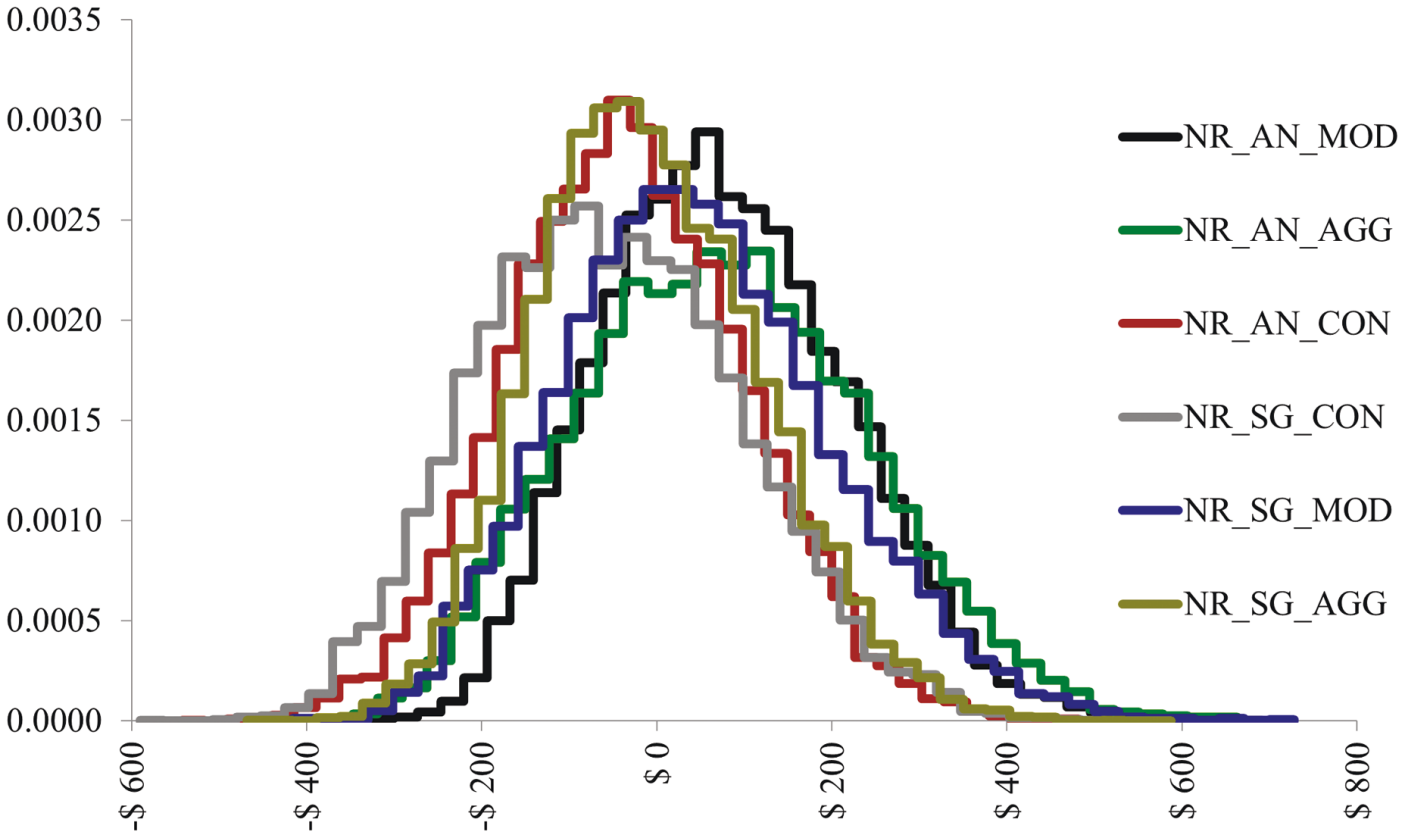
Simulated Probability Density Functions for each Treatment Group Net Return.

The results of simulated NR for each treatment group are summarized in Table 3. The simulated cumulative distribution functions and probability density functions for each of the treatment groups are contained within Figures 1 and 2, respectively.

**Table 3.** Simulated net return summary statistics for each treatment group using the average pricing grid

Treatment group	Mean	Standard deviation	Minimum	Maximum
AN-CON	–US$33.49	US$134.83	– US$542.49	$481.95
AN-MI	US$78.70	US$137.11	– US$432.47	$627.76
AN-HI	US$75.84	US$163.55	$431.02	$691.00
SG-CON	–US$53.14	US$149.36	−$590.77	$512.76
SG-MI	US$47.03	US$151.15	−$415.93	$728.17
SG-HI	–US$6.98	US$128.00	−$468.29	$588.18

Notes: the grid used for pricing premiums and discounts of quality and yield grade was Prime = +US$19.54, Choice= +US$0, Select = –US$17.92, Standard = –US$31.50, Yield Grade 1 = +US$3.69, Yield Grade 2= +US$1.58, Yield Grade 3 = $0, Yield Grade 4 = –US$11.23, and Yield Grade 5= –US$16.85.

Looking at the mean net returns for the treatment groups in Table 3 suggests the expected net return per head for AN-MI>AN-HI>SG-MI>SG-HI>AN-CON >SG-CON. Figure 1 reveals AN-MI and AN-HI have the greatest probability of yielding the highest net returns. This is expected as the combination of higher expected ADG and marbling (i.e., quality grade) associated with these treatment groups would result in higher expected net return. A closer look at Figure 1 demonstrates the increased downside and upside risk associated with AN-HI as compared to AN-MI. We would expect the probability of negative net returns per head under the AN-MI treatment group to be 30.4% while the AN-HI group would be 34%. However, the upside risk is also increased for the AN-HI treatment group resulting in the probability of net return per head exceeding US$200 being 22.8% for that group as compared to 19.5% for the AN-MI group. Within Figure 2, a comparison of the PDFs for the treatment expected net returns reveals overall similar looking distributions between treatments with AN-MI and AN-HI displaying greater likelihood of increased net returns as compared to the other treatments on average.

**Figure 3.**
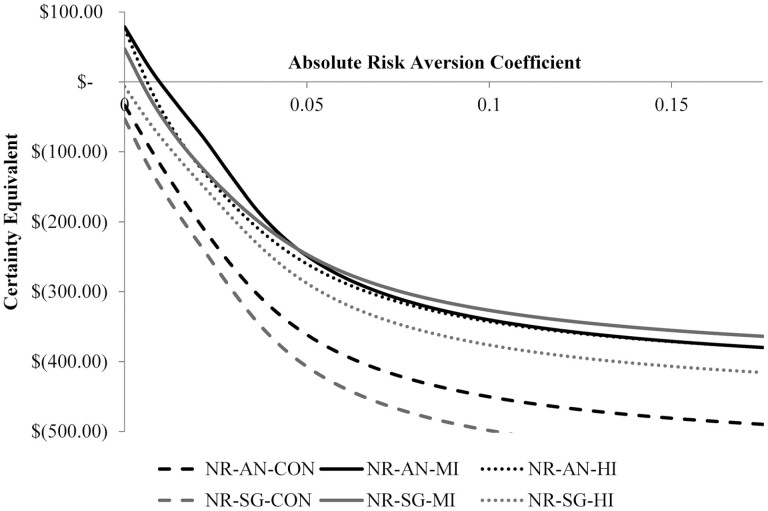
Stochastic Efficiency with Respect to a Function (SERF) Under Negative Exponential Utility Function Comparing the Simulated Net Returns per Head of Each Treatment Group.

To provide an objective ranking of the treatment groups while considering differing risk appetites of potential feedlot managers, we conduct an SERF analysis (stochastic efficiency with respect to a function). SERF is a method of stochastic dominance with respect to a function (SDRF) that allows for ranking of a set of risky alternatives in terms of their expected certainty equivalents (CE) for a specified range of attitudes to risk. A CE is the sure amount that the decision maker would view as equally desirable as compared to a specific risky alternative. The main advantage of SERF is that it allows each alternative to be compared simultaneously to all other alternatives, which can produce a smaller efficient set than the traditional pairwise comparison of SDRF (Hardaker et al., 2004). Though SERF can be applied for any utility function for which the inverse function can be calculated, we assume a negative exponential utility function as suggested by Hardaker et al. (2004), in part, for its useful CARA (constant absolute risk aversion) property. McCarl (1990) demonstrated that the CARA function yields similar results as other utility functions over small risk aversion intervals lending additional support for its use in the current analysis. The results of the SERF analysis are displayed in Figure 3.

Through examination of Figure 3, the efficient set is determined to include treatment groups AN-MI and SG-MI depending on the assumed absolute risk aversion coefficient (ARAC) of the feedlot manager. The ARAC represents a decision maker’s degree of risk aversion with three general classifications indicated by the ARAC value: 1) risk averse if ARAC > 0, 2) risk neutral if ARAC = 0, and 3) risk preferring if ARAC < 0. The ARAC values used in this analysis ranged from 0 (risk neutral) to 0.2 (relatively strong risk aversion). We chose not to conduct the analysis for any level of risk aversion < 0 (risk preferring/seeking) as we assume producers are generally not risk seekers as suggested by the literature (Feuz et al., 1995; Bar-Shira et al., 1997). The efficient set within SERF analysis contains the options that are found to provide the highest certainty equivalent across the range of ARAC evaluated. By looking at Figure 3, the efficient set within this current analysis would contain only AN-MI for managers with an ARAC of >0 and <0.0458 and only SG-MI for managers with an ARAC of >0.0458. However, as the CE of the AN-MI treatment group for any manager with an ARAC of >0.0096 would be expected to be negative, the efficient set can be updated in practice to only include AN-MI. CE values provide useful information regarding the value of an option at a specified level of risk aversion. As an example, a CE of $8.96 for the AN-MI treatment for a relatively risk averse feedlot manager with an ARAC of 0.0083 suggests that if the manager was given the option of a guaranteed net return (revenue less feed and treatment costs) of US$8.96/hd., the manager would be indifferent between the guaranteed return and the risky alternative of producing and marketing AN-MI cattle. A negative value for a CE suggests that a manager would rather give up (pay) money rather than engage in the risky alternative. Thus, we exclude any treatment from the efficient set if the CE values are negative as we assume that the manager would choose not to produce the cattle at that level of risk aversion.

### Hypothetical Price Grid

The simulated results presented previously rely on a single price grid (USDA, 2021) from a single point in time. Using this pricing grid results in a good comparison of what we might expect the net returns for each treatment group to be assuming a similar price grid were used in reality. It is important to recognize, however, that feedlots may be paid on a grid that could be substantially different from the grid used in this analysis. As discussed previously, BI cattle have some favorable characteristics (less water consumption, more heat tolerant, more adaptive to nutrient stress, etc.) over BT cattle. However, this study demonstrates that on average the BI influenced treatment groups (SG-CON, SG-MI, and SG-HI) are expected to result in decreased net return per head compared to the angus counterpart. If feedlot managers are profit maximizers, when might we expect BI influenced steers to pay off? Two plausible scenarios are 1) when the price grid a feedlot is paid on favors yield grade improvements at a greater rate compared to quality grade, and 2) when the climate of a feedlot’s area favors the benefits of BI cattle.

The results of Reichhardt et al. (In Review) demonstrated that the angus treatment groups resulted in higher marbling percentage (quality grade), while the SG cattle had higher average cold camera yield grades as compared to AN. Though the differences in yield grade between the two breeds were not statistically significant in this current study, there is some evidence that BI influenced steers have increased yield grades as compared to BT steers (Reichhardt et al., 2021). Therefore, a grid favoring yield grade improvement at a greater rate than quality grade could possibly result in SG and AN cattle performing equally well in terms of the net return. Using a dynamic optimization model within Palisade’s @Risk (2019), we solve for strictly hypothetical price premiums and discounts for quality and yield grade that would result in AN-MI and SG-MI having similar expected net returns per head (NR_AN-MI_ – NR_SG-MI_ = 0). These two treatment groups are chosen for the break-even price grid analysis as they were the treatment groups from each breed with the expected highest net return. The solution to the dynamic model resulted in a hypothetical price grid as summarized in Table 2. The hypothetical grid solved for is only one of many such grids that may result in a break-even change in net return between SG-MI and AN-MI. Our objective is not to determine all such grids but by identifying one example break-even grid, we can compare it with the original grid used in the main analysis to determine what type of changes to the grid a producer would need to expect in general to result in a break-even condition between these two treatments.

**Figure 4.**
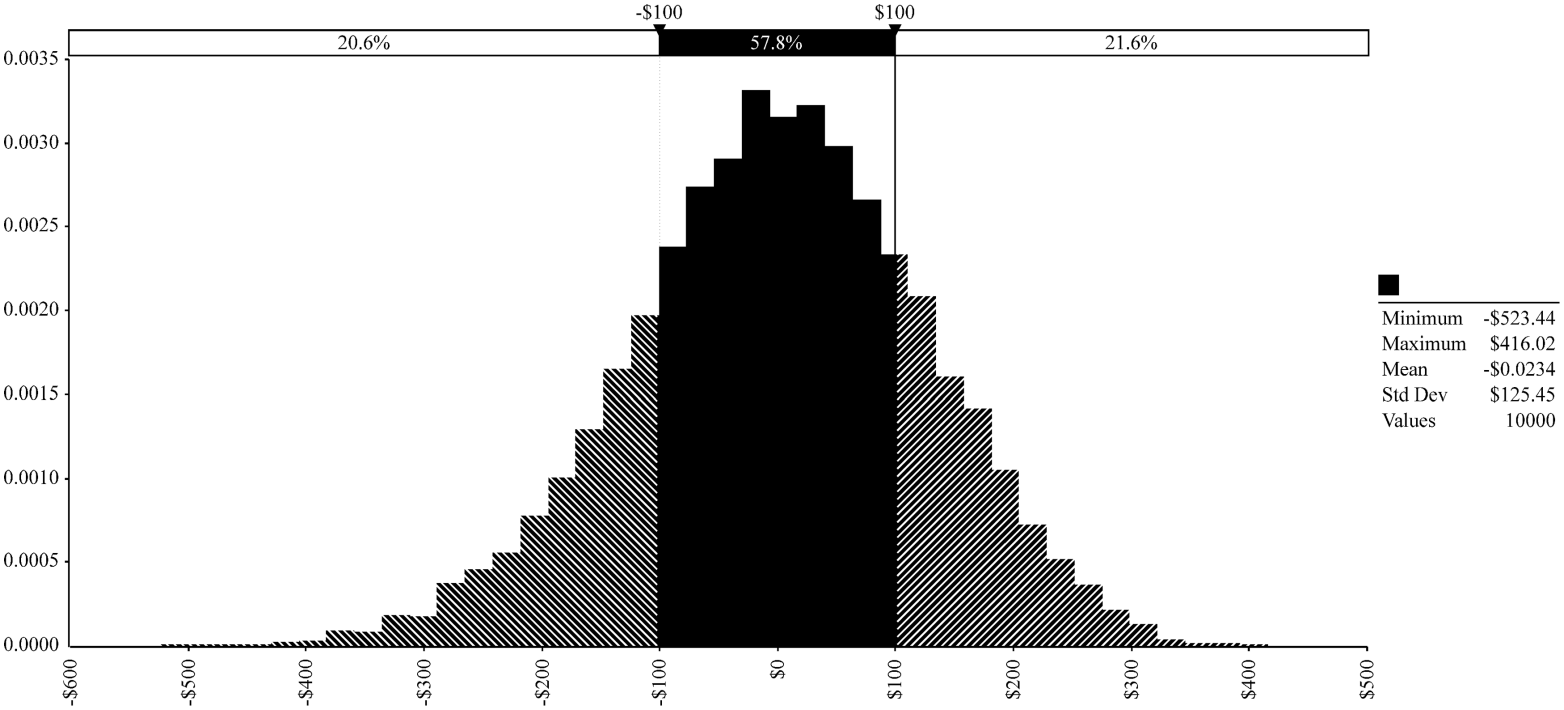
Probability Density Function of Simulated Difference in Net Return between AN-MI and SG-MI Using Hypothetical “Break-even” Price Grid.

Using the hypothetical “break-even” price grid (Table 2), the difference between NR_AN-MI_ and NR_SG-MI_ was simulated. The simulated PDF of this difference is contained within Figure 4. The simulated mean change in net return between these two treatments was US$0.02/hd. This result demonstrates that the break-even grid would result in nearly equal expected mean net return per head for these two treatment groups. The hypothetical price grid demonstrates that if yield grades 2 and 1 were favored over yield grade 3 by approximately US$6.71/cwt and US$14.90/cwt, respectively and quality grades “select” and “standard” discounted by –US $17.31/cwt and –US$22.65/cwt relative to “choice” it would be reasonable to expect AN-MI and SG-MI to perform similarly in expected net return per head. Notice in Table 2 that comparatively, the hypothetical grid values yield grade at a higher marginal rate whereas quality grade is valued at a lower marginal rate relative to the original price grid used in the simulation analysis. Whether this type of hypothetical grid is obtainable in the market is not investigated in this paper. However, the hypothetical grid is useful in demonstrating the expected convergence in net return per head between SG-MI and AN-MI as premiums for each increasing level of yield grade are rewarded more generously and quality grade less generously.

### Limitations

SG cattle have been shown to be more heat tolerant with decreased water consumption as compared to Angus (Winchester and Morris, 1956; Forbes et al., 1998; Beatty et al., 2006). Therefore, we might expect that when produced in a region characterized by a hot climate, SG cattle would have advantages in production as compared to AN, holding all else constant. This study was conducted in Northern Utah during the months of October to May when high temperatures would be of little concern. When evaluating the results from this study, it is important to recognize that due to the region and time of year in which the study took place, the production advantages of SG cattle (specifically heat tolerance) would presumably have a decreased effect on the calculation of NR.

Additionally, these results are based on the data from one relatively small study with two Angus influenced samples, with even the SG influenced treatment group having a majority (50%) Angus influence in their breed type. Therefore, the results presented could be strengthened through additional research including enlarging the sample size and increasing the amount of BI influence. The results provide a good baseline upon which additional research efforts surrounding this objective can build.

## CONCLUSION

This study evaluates the expected net return per head of SG influenced steers and Angus steers with varying levels of anabolic implant. AN-MI steers are expected to have the highest average net return (US$78.70/hd.) followed by AN-HI (US$75.84/hd.), SG-MI (US$47.03/hd.), SG-HI (–US$6.98/hd.), AN-CON (–US$33.49/hd.), and SG-CON (US$53.14/hd). While the simulated net returns for AN-MI and AN-HI were similar, the decreased risk (lower standard deviation) associated AN-MI makes it the optimal choice from an economic perspective for steers in the study region. The AN-MI treatment group would also be preferred to the AN-HI by feed yard management when considering the decreased time investment in the moderate implant protocol as compared to the aggressive protocol. SERF analysis confirms that when the CE values are limited to those ≥US$0, only AN-MI remains in the efficient set.

The results are based on a small sample from a study in a cooler climate, using a single price grid from one point in time. Feedlots within relatively hotter climates may see additional benefits to using *Bos Indicus* influenced cattle as they have been shown to be more heat tolerant as compared to *Bos Taurus* cattle. The difference in expected net return per head of AN-MI and SG-MI is US$31.67 (Figure 1). Thus, when it comes to breed selection, as long as feedlot managers anticipate at least a US$32.00/head benefit in their region from using SG influenced cattle, this selection could be financially justified over AN. Additionally, managers must remain cognizant of the expected premiums/discounts they routinely receive from the packer. The AN steers in this study were shown to have increased marbling grades compared to SG, whereas the SG steers averaged lower yield grade (increased yield) as compared to the Angus steers. Thus, the more a price grid favors increased yield as opposed to quality, the better we might expect SG steers to perform economically as compared to AN.

## Supplementary Material

txac111_suppl_Supplementary_Material
