# Supplementary material for: Comparing net returns in the feedlot: Bos Taurus vs. Bos Indicus influenced steers with varying anabolic implant intensity
Source: Transl Anim Sci. 2022 Aug 21;6(3):txac111. doi: 10.1093/tas/txac111 (PMC9512098; doi:10.1093/tas/txac111)
Supplement: txac111_suppl_Supplementary_Material [file txac111_suppl_supplementary_material.docx]

**APPENDIX 1: SUMMARY OF DISTRIBUTIONS USED IN SIMULATION ANALYSIS**

| **Table A1. Distributions Fit for Stochastic Variables in Simulation of Treatment Group Net Returns** | | | | | |
| --- | --- | --- | --- | --- | --- |
| **Variable** | **Graph** | **Function** | **Min** | **Mean** | **Max** |
| Fed Cattle Price ($/cwt) |  | Weibull | 152.41 | 183.89 | +∞ |
| Alfalfa ($/ton) |  | Triangle | 116.40 | 167.35 | 206.66 |
| Barley ($/bu.) |  | Laplace | -∞ | 3.16 | +∞ |
| Corn ($/bu.) |  | Loglogistic | 3.08 | 3.70 | +∞ |
| AN-CON Average Daily Gain (lbs./day) |  | Triangle | 1.96 | 2.61 | 3.15 |
| AN-MI Average Daily Gain (lbs./day) |  | Triangle | 3.09 | 3.37 | 3.67 |
| AN-HI Average Daily Gain (lbs./day) |  | Triangle | 2.99 | 3.62 | 4.25 |
| SG-CON Average Daily Gain (lbs./day) |  | Triangle | 1.95 | 2.77 | 3.52 |
| SG-MI Average Daily Gain (lbs./day) |  | Triangle | 3.01 | 3.62 | 4.23 |
| SG-HI Average Daily Gain (lbs./day) |  | Triangle | 2.87 | 3.43 | 3.89 |
| AN-CON Dressing % |  | Triangle | 0.55 | 0.60 | 0.65 |
| AN-MI Dressing % |  | Triangle | 0.58 | 0.61 | 0.65 |
| AN-HI Dressing % |  | Triangle | 0.58 | 0.61 | 0.65 |
| SG-CON Dressing % |  | Triangle | 0.56 | 0.60 | 0.63 |
| SG-MI Dressing % |  | Triangle | 0.57 | 0.61 | 0.64 |
| SG-HI Dressing % |  | Triangle | 0.58 | 0.60 | 0.63 |
| Note: While not displayed in this table, stochastic variables for “as fed intake” (lbs./day) for the 5 step up rations per treatment group were also created using simple triangle distributions fitted to the intake data collected.  The variables for USDA yield and quality grade both relied on separate uniform distributions to select random observations from within a breed and treatment with the actual yield and quality grade for the corresponding observations used for each simulation iteration. | | | | | |

*Distributions*

All distributions for the variables that relied on historical data were fit using Palisades @Risk Decision Tools Suite 7.6 (2019) with the distributions selected according to ‘best’ fit ranked by Akaike information criterion (AIC). These variables included *Fed Cattle Price*, *Alfalfa* price, *Barley* price, and *Corn* price. Fitted distributions were relied on for these instances as the sample size for each variable was large allowing for a good overall fit of distributions. All other variables formed from the data collected during the feeding trial, except for USDA quality and yield grade, relied on triangle distributions. These variables included treatment ADG, treatment dressing percentage, and treatment ration as feed intakes. Triangle distributions are useful when a distribution’s shape can only vaguely be identified, but where upper and lower limits can be reasonably estimated along with a ‘best guess’, regarding the most likely outcome (Fairchild, Misra, and Shi, 2016). This is the situation with our feeding trial variables. The limited sample size (noted in the limitation section) makes it difficult to fit distributions using the AIC. The parameters of the triangle distributions used the range of each variable in the trial to set the minimum and maximum values, with the averages used as the most likely value. The USDA quality and yield grade variables relied on uniform distributions to select random observations from within a breed and treatment with the actual yield and quality grade for the corresponding observations used for each simulation iteration. This was done for two reasons. First, the limited sample size again, prevented fitting distributions according to the AIC and second, the distributions are composed of discrete values making the use of a triangle distribution in these cases an inappropriate option. We wanted the analysis to be informed by the sample data as best we could with the limited sample size. Thus, we used the uniform distribution in the case of quality and yield grade to randomly select one of the actual values of observed quality and yield grade by treatment within each simulation iteration.

**Reference**

Fairchild, K. W., L. Misra, and Y. Shi. 2016. Using triangular distribution for business and finance simulations in Excel. J. Fin. Edu. 42:313–336. https://www.jstor.org/stable/90001156
